# Mutation Analysis of *Inhibitory Guanine Nucleotide Binding Protein Alpha* (*GNAI*) Loci in Young and Familial Pituitary Adenomas

**DOI:** 10.1371/journal.pone.0109897

**Published:** 2014-10-07

**Authors:** Hande Demir, Iikki Donner, Leena Kivipelto, Outi Kuismin, Camilla Schalin-Jäntti, Ernesto De Menis, Auli Karhu

**Affiliations:** 1 Department of Medical Genetics, Genome-Scale Biology Research Program, Institute of Biomedicine, Biomedicum Helsinki, University of Helsinki, Helsinki, Finland; 2 Department of Neurosurgery, Helsinki University Central Hospital, Helsinki, Finland; 3 Department of Clinical Genetics, Oulu University Hospital, Oulu, Finland; 4 Division of Endocrinology, Department of Medicine, Helsinki University Central Hospital, Helsinki, Finland; 5 Department of Internal Medicine, General Hospital, Montebelluna, Treviso, Italy; National Cancer Institute, United States of America

## Abstract

Pituitary adenomas are neoplasms of the anterior pituitary lobe and account for 15–20% of all intracranial tumors. Although most pituitary tumors are benign they can cause severe symptoms related to tumor size as well as hypopituitarism and/or hypersecretion of one or more pituitary hormones. Most pituitary adenomas are sporadic, but it has been estimated that 5% of patients have a familial background. Germline mutations of the tumor suppressor gene *aryl hydrocarbon receptor-interacting protein* (*AIP*) predispose to hereditary pituitary neoplasia. Recently, it has been demonstrated that *AIP* mutations predispose to pituitary tumorigenesis through defective inhibitory GTP binding protein (Gα_i_) signaling. This finding prompted us to examine whether germline loss-of-function mutations in *inhibitory guanine nucleotide (GTP) binding protein alpha* (*GNAI*) loci are involved in genetic predisposition of pituitary tumors. To our knowledge, this is the first time *GNAI* genes are sequenced in order to examine the occurrence of inactivating germline mutations. Thus far, only somatic gain-of-function hot-spot mutations have been studied in these loci. Here, we have analyzed the coding regions of *GNAI1*_,_ *GNAI2*, and *GNAI3* in a set of young sporadic somatotropinoma patients (*n* = 32; mean age of diagnosis 32 years) and familial index cases (*n* = 14), thus in patients with a disease phenotype similar to that observed in *AIP* mutation carriers. In addition, expression of Gα_i_ proteins was studied in human growth hormone (GH), prolactin (PRL), adrenocorticotropic hormone (ACTH)-secreting and non-functional pituitary tumors. No pathogenic germline mutations affecting the Gα_i_ proteins were detected. The result suggests that loss-of-function mutations of *GNAI* loci are rare or nonexistent in familial pituitary adenomas.

## Introduction

Pituitary adenomas are neoplasms of the anterior pituitary lobe. They account for 15–20% of all the intracranial tumors [1] and approximately 16% of all the primary brain and central nervous system tumors [2]. The hallmarks of pituitary tumors are hormonal dysfunction, i.e hormonal hypersecretion or hypopituitarism and local symptoms related to the tumor mass. Compression of neighboring structures may cause headaches and visual impairment [3]. Pituitary adenomas are classified based on the pituitary cell of origin and the type of hormone secreted. The most common functional pituitary tumors hypersecrete prolactin (PRL) (40–45%). Patients with prolactinomas present with amenorrhea, infertility and galactorrhea in females, and infertility in males. Somatotropinomas hypersecrete growth hormone (GH) (20–25%), causing acromegaly with clinical features of enlarged extremities, coarse facial structures and comorbidities such as hypertension, cardiovascular disease and diabetes mellitus [4]. The rate of mortality associated to untreated acromegaly has been reported to be two to four times higher than that seen in the healthy population [5], [6]. In many cases, slow progression of the symptoms delays the diagnosis [7]. Somatotropinoma during childhood or adolescence, before the growth of the long bones is complete, leads to gigantism. Tumors secreting adrenocorticotropic hormone (ACTH) (10–12%) cause Cushing’s disease, which is characterized by hypercortisolism. The majority of the other adenomas are non-functioning (non-secreting) pituitary adenomas (NFPA) [4]. All in all, pituitary adenomas cause a heavy clinical burden due to increased morbidity and the treatment modalities involved, i.e neurosurgery, chronic medical therapies and radiotherapy.

Most pituitary adenomas are sporadic but it has been estimated that 5% of affected patients have a familial background [8]. Pituitary adenomas occur as components of familial tumor syndromes such as multiple endocrine neoplasia type 1 (MEN1) [9], [10], Carney’s complex (CNC) [11], [12] and MEN4 [13]. Furthermore, in 2006, Vierimaa *et al.* found that germline mutations in the *aryl hydrocarbon receptor interacting protein* (*AIP*) gene cause pituitary adenoma predisposition (PAP) [14]. *AIP* mutations are mostly associated with somatotropinomas (78%), although cases with prolactinomas, NFPAs and Cushing’s syndrome have also been reported [15], [16]. The patients with *AIP* mutations are typically young (mean age at diagnosis 25 years) and do not necessary have a strong family history of the disease. *AIP* associated pituitary tumors are often large and invasive and resistant to the effects of available treatments, such as somatostatin analogues, which are used in acromegaly [17]–[19]. Familial occurrence of pituitary tumors is also the main feature in familial isolated pituitary adenoma (FIPA) [8], [20]. Subsequently, it was found that *AIP* germline mutations explain 15–20% of FIPA families and 50% of families with isolated familial somatotropinomas (IFS) [15]. Thus, the majority of FIPA families appear to be influenced by some other, as yet unidentified genes responsible for familiar clustering of pituitary tumors. Identification of new predisposing genes would enable earlier detection of pituitary adenomas and contribute to clinical management of patients.

The *stimulatory guanine nucleotide (GTP) binding protein alpha* (*GNAS*; encoding Gα_s_ subunit) has been found to be mutated in 30–40% of sporadic somatotropinomas. These somatic gain-of-function mutations lead to constitutive activation of cyclic adenosine monophosphate (cAMP) synthesis and increased proliferation through cAMP mediated mitogenic signaling [21]–[24]. Activating mutations on *GNAS* are also associated to McCune-Albright syndrome [25], [26]. Along with well-established *GNAS* mutations, somatic mutations in other Gα family members, namely *GNAQ* and *GNA11*, have been linked to tumorigenesis in melanocytic neoplasms [27], [28].

We have recently demonstrated that *AIP* loss-of-function mutations predispose to pituitary tumorigenesis through defective inhibitory GTP binding protein (Gα_i_) signaling and consequent elevated intracellular cAMP concentrations [29]. We found that Gα_i-2_ and Gα_i-3_ proteins are not capable of inhibiting cAMP synthesis during *AIP* deficiency and that Gα_i-2_ protein levels are significantly reduced in *AIP*-mutated somatotropinomas. As the AIP protein seems to be an essential regulator of Gα_i_ signaling, the possibility that inactivating germline mutations in *GNAI* loci (encoding Gα_i_ subunits) would predispose to pituitary adenomas prompted us to investigate the role of these genes in pituitary tumorigenesis. Here we sequenced all the coding exons of *GNAI1*_,_ *GNAI2* and *GNAI3* in a set of young sporadic somatotropinoma patients and familial index cases, thus in patients with a disease phenotype similar to that observed in *AIP* mutation carriers.

## Materials and Methods

### Gα_i_ immunohistochemistry

To investigate the expression of Gα_i_ proteins in human pituitary tumors, Gα_i-1_, Gα_i-2_ and Gα_i-3_ immunostainings were performed in four prolocatinomas, six somatotropinomas, three ACTH and four NFPA tumors. All tumors were *AIP* mutation negative. Antibodies used were mouse monoclonal antibody against Gα_i-1_ (SPM397, sc-56536, Santa Cruz, 1: 40), rabbit polyclonal antibody against Gα_i-2_ (T19, sc-7276, Santa Cruz, 1: 60) and mouse polyclonal antibody against Gα_i-3_ (H00002773-B01P, Abnova Corp. Taipei city, Taiwan, 1: 50). Anti-mouse/rabbit/rat secondary antibody, Poly-HRP-GAM/R/R (DPVB55HRP, Immunologic, Duiven, Netherlands) and DAB chromogen (Lab Vision Corporation, Fremont, CA, USA, Thermo Fisher Scientific, Watham, MA, USA) were used for detection. Immunostaining protocol was applied as described [30]. The staining intensities of Gαi proteins were scaled as negative (0), weak (1), moderate (2), or strong (3). The images were taken and edited by Leica DM LB microscope (Meyer Instruments, Houston, TX, USA), Olympus DP50 camera (Olympus Corporation, Tokyo, Japan) and Studio Lite software (Licor, Lincoln, NE, USA).

### Patients

This study included a set of 32 young sporadic GH-secreting pituitary adenoma cases in which three of the tumors were secreting both GH and PRL. Age at diagnosis for sporadic cases ranged from 14 to 56 years with a mean of 32 years (Table 1). A majority of the tumors were macroadenomas. The second set of samples included 14 index cases with a familial history of pituitary adenomas (Table 1). The hormones secreted by the tumors were GH (*n* = 11), PRL (*n* = 1), ACTH (*n* = 1) and NFPA (*n* = 1). All the patients had previously been sequenced negative for *AIP*. From familial cases 9/14 were earlier screened negative for large germline deletions of *AIP* [31]. The study and the consent procedures were approved by the Ethics Committee of the Hospital district of Helsinki and Uusimaa (HUS) (approval number: 408/13/03/03/2009) and the Institutional Review Board of the Department of Internal Medicine, General Hospital, Montebelluna (Treviso). Signed informed consent was obtained from all the study participants. In case of the minor/children, the consent was obtained from parent/guardian. Consents are stored and managed together with patient information in the central office/ambulatories where the access is restricted.

**Table 1:** Patient information and variants detected in the coding regions of *GNAI* loci.

Patient	Sex	Age at Dg	Age at Op	Origin	Clinical Dg	TumorSize	Affectedfamilymember(s)	*GNAI1*	*GNAI2*	*GNAI3*
**S1**	M	37	–	Spain	GH	Macro	–	–	–	–
**S2**	M	40	–	Tunisia	GH	Macro	–	–	–	–
**S3**	F	38	–	Finland	GH	Macro	–	–	–	–
**S4**	M	14	–	Finland	GH	Macro	–	–	–	–
**S5**	F	24	–	Italy	GH/PRL	NA	–	–	–	–
**S6**	F	24	–	Italy	GH/PRL	Macro	–	–	–	–
**S7**	F	23	–	Italy	GH	Macro	–	–	–	–
**S8**	M	22	–	Italy	GH	NA	–	–	–	–
**S9**	F	19	–	Italy	GH	Macro	–	–	–	–
**S10**	F	17	–	Italy	GH	Macro	–	–	–	–
**S11**	M	33	–	Italy	GH	Macro	–	c.468G>GA(rs12721456)	–	c.105G>GA(rs2230350) c.987G>GA(rs61758987)
**S12**	M	30	–	Italy	GH	Macro	–	c.468G>GA(rs12721456)	c.138C>CT(rs762707)	–
**S13**	F	37	–	Italy	GH	Macro	–	c.846T>TC(rs10241877)	–	–
**S14**	F	36	–	Italy	GH	Micro	–	–	–	–
**S15**	F	33	–	Italy	GH	Macro	–	–	–	–
**S16**	M	36	–	Italy	GH	Macro	–	–	–	–
**S17**	M	23	–	Italy	GH	Macro	–	–	–	–
**S18**	M	35	–	Italy	GH	Macro	–	c.846T>TC(rs10241877)	–	–
**S19**	F	32	–	Italy	GH	Macro	–	–	–	–
**S20**	F	36	–	Italy	GH	Macro	–	c.468G>GA(rs12721456)	c.138C>CT(rs762707)	–
**S21**	M	39	–	Italy	GH	Macro	–	–	–	(c.105G>GA)rs2230350
**S22**	M	38	–	Italy	GH	Micro	–	c.468G>GA(rs12721456)	–	–
**S23**	M	26	–	Finland	GH	NA	–	c.846T>TC (rs10241877)	–	–
**S24**	F	40	–	Italy	GH	NA	–	–	–	–
**S25**	M	23	–	Finland	GH/PRL	Macro	–	c.846T>TC(rs10241877)	–	–
**S26**	F	43	–	Finland	GH	NA	–	c.846T>TC(rs10241877)	–	–
**S27**	F	24	–	Finland	GH	Macro	–	–	–	–
**S28**	F	39	–	Estonia	GH	Macro	–	–	–	–
**S29**	M	40	–	Finland	GH	NA	–	–	–	–
**S30**	F	25	–	Italy	GH	NA	–	–	–	–
**S31**	M	56	–	Finland	GH	NA	–	c.846T>TC(rs10241877)	–	–
**S32**	F	55	–	Finland	GH	NA	–	–	–	–
**F1**	F	40	–	Italy	GH	Micro	NFPP (father)	–	–	–
*F2	F	56	NA	Italy	GH	NA	GH (aunt)	–	–	–
*F3	M	56	NA	Italy	NFPA	NA	GH (mother)	–	–	–
*F4	F	NA	67	Italy	ACTH	NA	GH (son)	–	–	–
*F5	F	NA	36	Italy	PRL	NA	GH (aunt)	–	c.138C>CT(rs762707)	–
*F6	F	NA	49	Italy	GH	NA	PRL (daughter)	–	–	–
*F7	M	42	NA	Italy	GH	NA	GH (cousin)	–	–	–
*F8	M	36	–	Finland	GH	NA	GH (uncle)	c.846T>TC(rs10241877)	–	–
**F9**	F	NA	59	Finland	GH	NA	PRL (niece)	–	–	(c.105G>GA)rs2230350
*F10	M	NA	44	Italy	GH	NA	NFPA (niece)	c.468G>GA(rs12721456)	–	–
**F11**	M	24	NA	Italy	GH	NA	GH/PRL (sister)	–	–	–
**F12**	F	36	NA	Italy	GH	NA	GH (brother)	–	–	–
**F13**	F	63	NA	Finland	GH	NA	ACTH (cousin)	c.846T>TC(rs10241877)	–	–
*F14	M	40	NA	Finland	GH	Macro	PRL (cousin)	–	–	–

Dg: diagnosis, Op: operation, S: sporadic, F: familial, M: male, F: female, NA: not available, Micro: <10 mm, Macro: >10 mm. * Screened negative for *AIP* germline deletions by MLPA.

### Mutation Analysis on *GNAI* loci

The coding regions of *GNAI1* (ENST00000442586 and ENST00000351004; Ensemble release 75), *GNAI2* (ENST00000422163, ENST00000451956 and ENST00000266027), and *GNAI3* (ENST00000369851) were amplified and sequenced from blood-derived DNA. Also intronic regions flanking the exons were included in the analyses. PCR was carried out by mixing 0.25 µl 20 mM of each primers (Table 2), 5 ng/ul of DNA, 0.4 µl 40 mM of dNTP, 2.5 µl 10xPCR Buffer, and 0.1 µl AmpliTaq Gold DNA Polymerase (Invitrogen Life Science Technologies, Foster City, CA) in a final volume of 25 µl. PCR products were purified by using ExoSAP-IT PCR product cleanup reaction (Affymetrix, USB Products, CA, USA). DNA was sequenced by using BigDye v.3.1 sequencing chemistry and ABI3830x DNA sequencer (Applied Biosystems, Foster City, CA, USA). Sequences were analyzed with Mutation Surveyor software V4.0.8 (Soft-Genetics, State College, PA, USA).

**Table 2:** Primer sequences, annealing temperatures and Ensembl transcripts for 23 amplicons of *GNAI* loci.

Primer	Sequence (5′ –3′)	T_m_ (°C)	Trancript
Gα_i1__ex1_F	GGATTCCCCTGTGCTTGGA	60	ENST00000442586
Gα_i1__ex1_R	GTTTCCAAACGCCGAGGG		
Gα_i1__ex2&3_F	CACACAGAGAGAGACTGGGTG	60	ENST00000351004
Gα_i1__ex2&3_R	GGTCCTGATAGTTGACAAGCC		
Gα_i1__ex4_F	AAGGAAGTTCGCTATTGCC	60	ENST00000351004
Gα_i1__ex4_R	AATGTGTCAGCCAATTCTGC		
Gα_i1__ex5_F	GTTTTGGATGATCTTTATTGGC	60	ENST00000351004
Gα_i1__ex5_R	TCTCCCAAACATTCTTTTGTCC		
Gα_i1__ex6_F	CCCATAAAGTCCTTCTCTCCTTC	62×1, 61×1, 60×2, 59×2,58×2	ENST00000351004
Gα_i1__ex6_R	CTTGGCAACACCTTCAGCTC		
Gα_i1__ex7_F	TGTTCTGAAATGGCAGAAATG	60	ENST00000351004
Gα_i1__ex7_R	CTGAATTCTTGCCTTAGGGG		
Gα_i1__ex8_F	GGAGTCCATGAATGAAACTGTATG	60	ENST00000351004
Gα_i1__ex8_R	TTTGGTCAAGTCCCAGATGC		
Gα_i2__ex1c_F	TCACCCACATCACCGTCTAA	59	ENST00000422163
Gα_i2__ex1c_R	ACGCGTCCTCTTGCAACTA		
Gα_i2__ex1d_F	CGCTGTCCATTGCTCTTCAT	60	ENST00000451956
Gα_i2__ex1d_R	GCACATGTGAGCATTCAGGT		
Gα_i2__ex2_F	AGCTGAAGTGTGACGCTGTG	58	ENST00000266027
Gα_i2__ex2_R	CTTGGCCAGCCATGAAGG		
Gα_i2__ex3&4_F	ATGTGAGAACAGGGTGGCTC	58	ENST00000266027
Gα_i2__ex3&4_R	GGATTCCCTAGGATGAGACTTG		
Gα_i2__ex5_F	CCAAGAATACCCTAGCCTGG	60	ENST00000266027
Gα_i2__ex5_R	GCAAAGACCAGCAGTGTCC		
Gα_i2__ex6_F	CTACCTGAACGACCTGGAGCGTA	58	ENST00000266027
Gα_i2__ex6_R	CTCTGCTACCCCAGAGGCTG		
Gα_i2__ex7&8_F	AAATGGGGTAGAAAGCCTCC	58	ENST00000266027
Gα_i2__ex7&8_R	TGGTCACCATAGGCTACTTGG		
Gα_i2__ex9_F	CTTGCTGCACACGTAGGATG	58	ENST00000266027
Gα_i2__ex9_R	CGCTTAGTTCTTCCCCAGC		
Gα_i2__ex9b_F	GTCCACCTGCTCATTCTCGT	60	ENST00000266027
Gα_i2__ex9b_R	TGGAACCCAATTCTGTGGAG		
Gα_i3__ex1_F	GCAGTTTCCGTGGTGTGAG	58	ENST00000369851
Gα_i3__ex1_R	GTTCAGGCCTTCCAAGCG		
Gα_i3__ex2&3_F	TAGGACCCGTGGTTTTCATC	60	ENST00000369851
Gα_i3__ex2&3_R	TTGTTGCTTAAATTCATTTCCC		
Gα_i3__ex4_F	CTGGCCTGTCAGAAAAGGTC	60	ENST00000369851
Gα_i3__ex4_R	AAACATTTCCTTAAGTGGGGAC		
Gα_i3__ex5_F	TTTGCTATGCACATGGTTGG	60	ENST00000369851
Gα_i3__ex5_R	AAATTTTACCCTGATTAAGAGATGG		
Gα_i3__ex6_F	CATTTCAGTTTAGGGGAAGGTG	60	ENST00000369851
Gα_i3__ex6_R	TTATTTTCCATTTCCTGGCTAC		
Gα_i3__ex7_F	TGAATGCCATTTAGTGCTGC	60	ENST00000369851
Gα_i3__ex7_R	GCCACTACCACTGAATACTCTCC		
Gα_i3__ex8_F	TTGGGTTATGTTCCCTCTCC	60	ENST00000369851
Gα_i3__ex8_R	CAAGAGACATCACTGTAGCACTATAAC		

T_m_: annealing temperature.

## Results

### Gα_i_ immunohistochemistry

**Figure 1:**
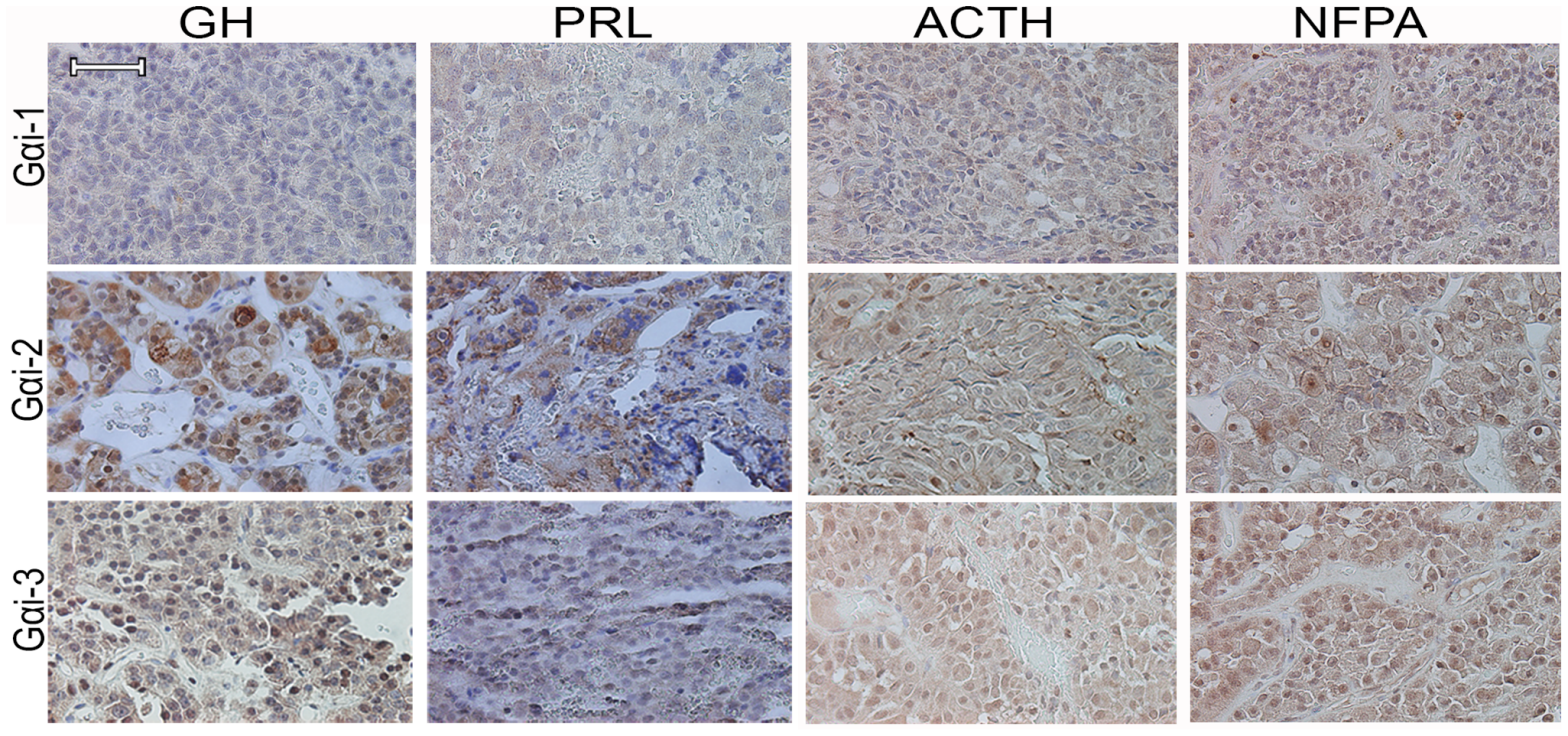
Gα_i-1,_ Gα_i-2_, and Gα_i-3_ protein expressions in GH, PRL, ACTH and non-functioning (NFPA) pituitary adenomas. Scale bar = 20 µm.

To examine the Gα_i_ protein expressions in human pituitary adenomas, Gα_i-1,_ Gα_i-2,_ Gα_i-3_ expressions were immunohistochemically (IHC) analyzed in *AIP* mutation negative somatotropinomas, prolactinomas, NFPA and ACTH tumors. Weak and speckled cytoplasmic expression of Gα_i-1_ was detected in GH- (mean±SD; 0.8±0.4) and PRL- (1±0.8) secreting tumors, whereas NFPA (1.8±0.5) and ACTH (1.7±0.6) tumors showed weak to moderate cytoplasmic expression (Figure 1). Consistent with the earlier observation in human GH-secreting tumors [29], Gα_i-2_ was prominently expressed in the cytoplasm of the somatotropinomas (2.8±0.4). Prolactinomas displayed moderate to strong expression of Gα_i-2_ (2.5±0.6). NFPA (1.8±0.5) and ACTH (1.7±0.6) adenomas showed moderate cytoplasmic and occasional nuclear Gα_i-2_ staining. All tumor types displayed moderate cytoplasmic expression of Gα_i-3_ (GH: 2.3±0.5, PRL: 2±0.8, ACTH: 1.6±0.6, NFPA: 1.8±0.5). Weak to moderate nuclear Gα_i-3_ staining was also observed in all tumor types (GH: 1.3±0.8, PRL: 0.8±0.5, ACTH: 1.3±0.6, NFPA: 1.5±0.6).

### *GNAI* loci mutation analysis

All the *GNAI* coding exons (23 amplicons per sample) were successfully sequenced and analyzed in 32 young sporadic somatotropinoma and 14 index familial cases (Table 1). In *GNAI1*, earlier reported synonymous heterozygous variations were detected in exon 6 (rs12721456/5 samples) and in exon 7 (rs10241877/8 samples). In *GNAI2*, one reported heterozygous and synonymous variation was found in exon 4 (rs762707/3 samples). Also in *GNAI3,* only previously observed heterozygous synonymous variations were detected in exon 1 (rs2230350/3 samples) and exon 8 (rs61758987/1 sample). None of these variants modified amino acid sequence, indicating the polymorphic nature of these changes. Additionally, several reported and unreported variations were observed in intronic regions (Table S1). All the intronic variants located outside of the splice site consensus sequences and are thus not assumed to affect splicing events.

## Discussion

Many G proteins have been linked to tumor development, starting with the discovery that somatic gain-of-function mutations of codons 201 and 227 in the *GNAS* gene are responsible in one third of the sporadic somatotropinomas with elevated cAMP levels [23], [32]. Activating *GNAS* hot-spot mutations have been detected in many other tumor types. For instance, biliary tract, thyroid, pancreatic, colon, and testis tumors are common targets of somatic *GNAS* mutations. Additionally, activating somatic hotspot mutations have been reported in *GNAQ* (Gα_q_) and *GNA11* (Gα_11_) genes in melanomas and meningeal tumors [33]. Somatic mutations in other Gα subunit genes have been detected, albeit in a low frequency.

Proteins of the inhibitory Gα subfamily, Gα_i_/Gα_o_, mediate several cellular and metabolic functions [34]–[37]. Unlike the Gα_o_, Gα_i-1_, Gα_i-2_ and Gα_i-3_ subunits are involved in the hormonal inhibition of adenylate cyclase (AC) activity with subsequent decrease of intracellular cAMP levels [38], [39]. Previous studies have been focusing on screening *GNAI2* somatic hot-spot mutations (termed *gip2* oncogene) in codons 179 and 205. Somatic *gip2* mutations have been found in ovarian, adrenal, ACTH and NFPA tumors [32], [40], [41] **.** However, other studies have failed to confirm these initial findings [42]–[48]. Although isolated somatic mutations of *GNAI* genes have also been observed in next-generation sequencing efforts, further experiments are needed to validate the existence and relevance of these findings [49], [50].

In our original study, we found that *AIP* deficiency is associated in pituitary tumorigenesis via reduced Gα_i_ signaling followed by elevated cAMP concentrations [29]. In the current study, we searched for germline mutations in *GNAI* loci in pituitary adenoma patients compatible with the *AIP* phenotype; young patients with somatotropinoma and familial index cases (Table 1). Also protein expressions of Gα_i-1_, Gα_i-2_ and Gα_i-3_ were examined in human GH-, PRL-, ACTH- and non-secreting (NFPA) pituitary adenomas. We have earlier shown that Gα_i-2_ and Gα_i-3_ proteins are expressed in human somatotropinomas [29]. Here we observed that also the Gα_i-1_ protein, although at low levels, is present in GH-secreting pituitary adenomas. Moreover, immunoreactions against all three Gα_i_ proteins were detected in human prolactinomas, ACTH and NFPA tumors (Figure 1), suggesting a biological role of all these proteins in these tumor types as well.

We screened for germline mutations in the *GNAI* loci in sporadic somatotropinoma patients (*n* = 32) and familial index cases (*n* = 14) characterized by the *AIP* phenotype (Table 1). No pathogenic mutations were observed in any of the patients studied. All the detected variants were either known polymorphisms or located in intronic regions. Although certain intronic variants may cause impaired splicing, the observed variants were not proximal to known splice sites. We acknowledge that the sample size in the present study is insufficient to draw a definite conclusion of the involvement of *GNAI* germline mutations in genetic predisposition of pituitary tumors. Moreover, due to the small sample size there is no adequate power to detect possible associations between the observed variant alleles and a pituitary tumor phenotype.

To our knowledge, this is the first time that all the coding exons of *GNAI1*, *GNAI2* and *GNAI3* have been sequenced to detect germline loss-of-function mutations in a set of selected pituitary adenoma patients. All in all, our sequencing results suggest that germline mutations of the *GNAI* loci seem not to be associated to, or are rare in familial pituitary tumorigenesis. However, a larger set of samples, somatic mutation screenings, copy number profiling and additional cellular works would provide a more comprehensive result of the role of *GNAI* genes in pituitary tumorigenesis.

## Supporting Information

Table S1 Intronic variations in *GNAI* loci.
(DOCX)
