# Supplementary material for: Mutation Analysis of Inhibitory Guanine Nucleotide Binding Protein Alpha (GNAI) Loci in Young and Familial Pituitary Adenomas
Source: PLoS One. 2014 Oct 7;9(10):e109897. doi: 10.1371/journal.pone.0109897 (PMC4188600; doi:10.1371/journal.pone.0109897)
Supplement: Table S1 — Intronic variations in GNAI loci. (DOCX) [file pone.0109897.s001.docx]

**Table S1. Intronic variations in *GNAI* loci.**

| **Patients** | ***GNAI1*** | ***GNAI2*** |
| --- | --- | --- |
| **S1** | c.591-73T>C (rs3801356) | - |
| **S2** | - | - |
| **S3** | - | c.829+29A>AG (rs9830408) |
| **S4** | - | - |
| **S5** | - | c.-77G>GT; c.-76T>TA |
| **S6** | - | c.-77G>GT; c.-76T>TA |
| **S7** | - | c.-77G>GT; c.-76T>TA |
| **S8** | - | c.113+61C>CT (rs12721536) |
| **S9** | - | - |
| **S10** | - | - |
| **S11** | - | - |
| **S12** | c.591-73T>TC (rs3801356); c.721-78A>AG (rs10238438) | c.-77G>GT; c.-76T>TA; c.113+61C>CT (rs12721536) |
| **S13** | c.721-78A>AG (rs10238438) | - |
| **S14** | c.591-73T>TC (rs3801356); c.721-78A>AG (rs10238438) | - |
| **S15** | - | - |
| **S16** | - | - |
| **S17** | - | - |
| **S18** | c.591-73T>TC (rs3801356); c.721-78A>G (rs10238438) | - |
| **S19** | - | - |
| **S20** | - | - |
| **S21** | c.721-135T>TA | - |
| **S22** | - | - |
| **S23** | c.721-78A>AG (rs10238438) | - |
| **S24** | c.591-73T>TC (rs3801356) | - |
| **S25** | c.162-16C>CT; c.721-78A>AG (rs10238438) | - |
| **S26** | c.721-78A>AG (rs10238438) | - |
| **S27** | - | - |
| **S28** | - | - |
| **S29** | - | - |
| **S30** | c.591-73T>TC (rs3801356) | - |
| **S31** | c.591-73T>TC (rs3801356); c.721-78A>AG (rs10238438) | - |
| **S32** | - | - |
| **F1** | - | - |
| **F2** | c.591-73T>TC; c.721-78A>AG (rs10238438) | - |
| **F3** | - | c.113+61C>CT (rs12721536) |
| **F4** | - | - |
| **F5** | - | - |
| **F6** | c.591-73T>TC (rs3801356); c.721-78A>AG (rs10238438) | - |
| **F7** | c.119-72T>TG; c.721-78A>AG (rs10238438) | c.829+29A>AG (rs9830408) |
| **F8** | c.591-73T>TC (rs3801356); c.721-78A>AG (rs10238438) | - |
| **F9** | - | - |
| **F10** | - | - |
| **F11** | c.721-78A>AG (rs10238438) | - |
| **F12** | c.591-73T>TC (rs3801356) | - |
| **F13** | c.591-73T>TC (rs3801356) | - |
| **F14** | - | - |

S: sporadic, F: familial

All the unreported intronic variations were called based on the cDNA reference sequence of transcript ENST00000351004 for *GNAI1* and ENST00000266027 for *GNAI2* as described by Human Genome Variation Society (HGVS).
